# Foundations of Information Ethics

**DOI:** 10.5195/jmla.2020.829

**Published:** 2020-01-01

**Authors:** Gerald Natal

**Affiliations:** Raymon H. Mulford Health Science Library, University of Toledo, Toledo, OH, gerald.natal@utoledo.edu

As they become ever more pervasive, big technology companies such as Facebook, Google, and Amazon are being pressured to answer questions about how information collected on their platforms is being used and misused. Acts of cyber activism, such as the use of social media to speak out against government oppression, occur across the globe. Accusations of foreign interference in the 2016 US elections underscore the potential for damage to systems, individuals, nation-states, and humanity at large. “Fake news” has shaken confidence in the integrity of an impartial news media. *Foundations of Information Ethics* touches on these and related topics to pack a large amount of thought-provoking material in the 135 pages of the main sections of the book. The authors—educators, freelance researchers, and writers from the private sector—raise big questions concerning trust, limits, and the extent to which a society is willing trade freedoms for services.

As John T. F. Burgess points out in chapter one, lack of an online presence does not mean immunity to social, political, and economic forces that are subject to and affected by information misuse, a lack of transparency, and disinformation. A quote from former Secretary-General of the United Nations Kofi Annan in chapter two on telecommunications states that denial of services is “almost as acute” as a lack of jobs, food, shelter, health care, and drinkable water. In the same chapter, human rights are established as a framework for information ethics, and readers are presented with a good overview of how the handling of information impacts the rights of individuals.

The book also touches on the cosmopolitan nature of information systems and the challenges resulting from living in a pluralistic society, the effects of shifting generations, the ways big data can restructure society, and the hacker ethic. Each chapter serves to illustrate how the world has become populated with “global digital citizens,” exclusive of geographical and physical boundaries. All in all, readers should come away with a good sense of information ethics as an applied branch of knowledge that seeks to deal with constant change due to disruptive technologies.

The book introduces basic principles and history, and then launches into chapters highlighting the connections among ethics, information, and the various topics covered. Terminology, frameworks, and principles are provided to set the stage for explorations on access, privacy, data, intellectual property, cyber security, practical discourse, cognitive justice, and global digital citizenship. The final chapter concludes with a consideration of emerging and enduring issues in states of transmutation. Each chapter is loosely ordered, listing concepts, historical developments, influential figures, and main issues. Case studies, questions, and excerpts from primary sources are offered to stimulate thought and discussion. Extensive reference lists, additional resources for further investigation, and an occasional glossary accompany each chapter.

*Foundations of Information Ethics* should appeal to academics, students, practitioners, and anyone interested in the important topics at the intersection of ethics and information. Old problems are offered up for analysis, and new issues and concerns are brought to light. Several points are made throughout the book that have specific relevance to medicine and health care, specifically in reference to the use of big data, health data ownership, and the research data divide in medicine.

Also, anyone interested in the themes of power, social justice, decolonization, and democratization of knowledge should find this an enlightening read. Information professionals should come away with an idea of the positive role they can have in the area of human rights and information ethics. The book offers practical and conceptual viewpoints that serve as a wake-up call to be mindful of the ways in which data, information systems, and technology are abused and misused, reminding readers that security in the cyber world goes beyond information and information communication technologies and extends to people.

**Gerald Natal, MLIS, AHIP,** gerald.natal@utoledo.edu, Raymon H. Mulford Health Science Library, University of Toledo, Toledo, OH

